# Antiviral and immunological activity of zinc and possible role in COVID-19

**DOI:** 10.1017/S0007114521002099

**Published:** 2021-06-15

**Authors:** Dilina do Nascimento Marreiro, Kyria Jayanne Clímaco Cruz, Ana Raquel Soares de Oliveira, Jennifer Beatriz Silva Morais, Betânia de Jesus e Silva de Almendra Freitas, Stéfany Rodrigues de Sousa Melo, Loanne Rocha dos Santos, Bruna Emanuele Pereira Cardoso, Thaline Milany da Silva Dias

**Affiliations:** Departament of Nutrition, Federal University of Piaui, Campus Minister Petrônio Portela, Teresina, PI, Brasil

**Keywords:** Zinc, COVID-19, Antiviral, Immunomodulator

## Abstract

Zn deficiency compromises its biological functions, its effect on the immune system and its antiviral activity, increasing vulnerability to infectious diseases. This narrative review aims at presenting and discussing functional aspects and possible mechanisms involved in the potential role of Zn in the immune response and antiviral activity for coronavirus infectious disease-19 (COVID-19) prevention and control. The searches were conducted in PubMed and Science Direct databases, using clinical trials, experimental studies in animals and humans, case–control studies, case series, letters to the editor, and review articles published in English, without restrictions on year of publication. Search approach was based on using the terms: ‘zinc’, ‘COVID-19’, ‘antiviral agents’, ‘immunologic factors’ and ‘respiratory tract infections’. Literature shows the importance of Zn as an essential mineral immunomodulator with relevant antiviral activity in the body. Thus, although there is still a scarcity of studies evaluating Zn supplementation in patients with COVID-19, the results on the topic show the necessity of controlling Zn mineral deficiency, as well as maintaining its homoeostasis in the body in order to strengthen the immune system and improve the prevention of highly complex viral infections, such as that of the COVID-19.

Several micronutrients have been investigated worldwide to assess their role in the prevention and control of chronic diseases, such as diabetes mellitus, chronic kidney disease and cancer^(1–3)^. Zn, in particular, is an essential mineral involved in several biological processes, participating in the metabolism of carbohydrates, lipids and proteins, playing a relevant role as a cofactor, signalling molecule or structural element of biological components in the cells^(4,5)^.

Certain studies raised awareness of the relevance of Zn in the prevention of infectious diseases of the respiratory tract, like the coronavirus infectious disease-19 (COVID-19). Indeed, Zn is heavily investigated concerning its potential as a therapeutic target in the treatment of this infection because of performance of Zn in the immune response, regulating proliferation, differentiation, maturation and function of leucocytes and lymphocytes, as well as further modulating the inflammatory response^(6–9)^.

Furthermore, Zn has been shown to have an antiviral effect, inhibiting the interaction between the some virus and host cell and viral replication that could increase vulnerability to infectious diseases. The high prevalence of Zn deficiency worldwide should also be highlighted, mainly in high-risk groups including pre-mature babies and elderly, making them more susceptible to viral infection^(10,11)^.

Considering the importance of COVID-19 as an infectious disease of high prevalence and mortality rates, as well as the lack of clinical outcome that could identify the efficacy of Zn to the treatment of COVID-19, this narrative review aims at presenting and discussing in detail immunological aspects and possible mechanisms underlying effects of Zn in COVID-19 prevention and control.

## Methods

Our research focused on selecting studies that confirmed the role of Zn in COVID-19 prevention and control. The searches were conducted in the PubMed and Science Direct databases, in June 2020.We used clinical trials, experimental studies in animals and humans, case–control studies, series of cases, letters to the editor, and review articles published in English, without restrictions in the year of publication. The search strategy was based on using the terms: ‘zinc’, ‘COVID-19’, ‘antiviral agents’, ‘immunologic factors’ and ‘respiratory tract infections’. The keyword combinations used during the search for articles are shown in Table 1.

**Table 1.** Keyword combinations used in the search for articles*

1	Zinc AND (COVID-19 or 2019 novel coronavirus disease or COVID19 or COVID-19 pandemic or SARS-CoV-2 infection or COVID-19 virus disease or 2019 novel coronavirus infection or 2019-nCoV infection or coronavirus disease 2019 or coronavirus disease-19 or 2019-nCoV disease or COVID-19 virus infection)
2	Zinc AND (Antiviral Agents or Agents, Antiviral or Antivirals or Antiviral Drugs or Drugs, Antiviral) AND (Respiratory Tract Infections or Infection, Respiratory Tract or Respiratory Tract Infection or Infections, Respiratory or Respiratory Infections or Infections, Respiratory Tract or Upper Respiratory Tract Infections or Infections, Upper Respiratory Tract or Infections, Upper Respiratory or Respiratory Infection, Upper or Upper Respiratory Infection)
3	Zinc AND (Immunologic Factors or Immune Factors or Factors, Immune or Immunological Factors or Factors, Immunological or Factors, Immunologic or Immunomodulators or Biological Response Modifiers or Biomodulators or Modifiers, Biological Response or Response Modifiers, Biological) AND (Respiratory Tract Infections or Infection, Respiratory Tract or Respiratory Tract Infection or Infections, Respiratory or Respiratory Infections or Infections, Respiratory Tract or Upper Respiratory Tract Infections or Infections, Upper Respiratory Tract or Infections, Upper Respiratory or Respiratory Infection, Upper or Upper Respiratory Infection)

* Since the most articles included in this review were published before the COVID-19 pandemic, articles related to other respiratory diseases were included for a better understanding of involved mechanisms.

The research process was carried out independently by two authors and the articles included in the review were consensually selected. A third author was consulted in case of disagreement. In the first phase of the research, we analysed the title, summary, and keywords of the articles and identified those that met the eligibility criteria. The articles selected in the first phase were analysed by reading the full text and those eligible for the review were identified and included. In addition, we did manual search in reference list of eligible articles.

**Fig. 1.**
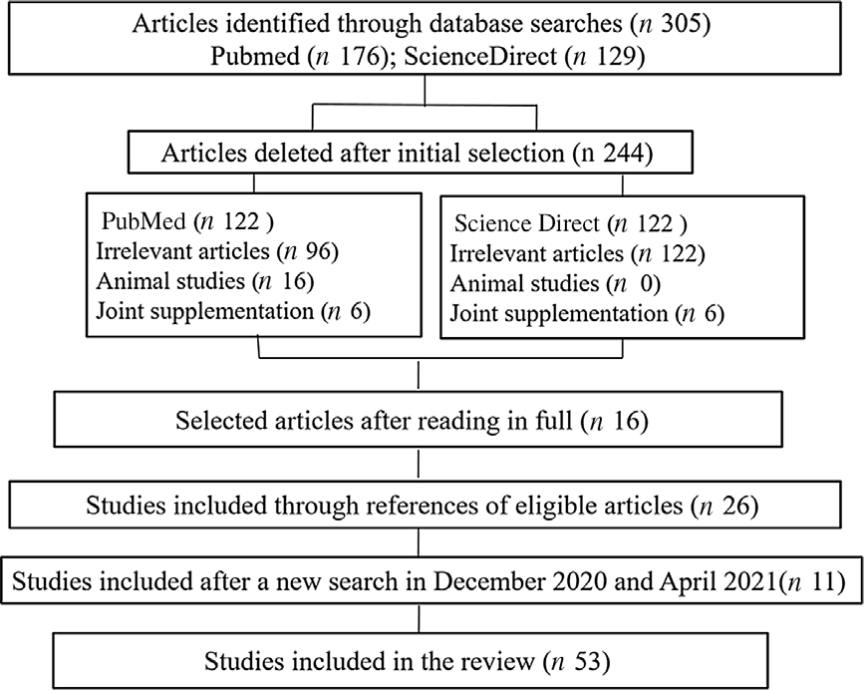
Diagram of study selection.

A total of 305 articles were identified by searching the PubMed (*n* 176) and Science Direct (*n* 129) databases. After the processes of selecting and removing duplicates, fifty-nine were identified as eligible based on the title and summary. After reading in full, sixteen studies were included. After that, twenty-five studies were included through the reference list search strategy of eligible articles, resulting in forty-one studies included in this review (Fig. 1). We conducted a new search in December 2020, and we included three more studies. Thus, this review included forty-four studies.

### COVID-19: antiviral activity and mechanism of action of zinc

Zn plays important physiological functions, in particular in neutralising the activity of several viruses, such as severe acute respiratory syndrome coronavirus 2 (SARS-CoV-2). This virus is part of the subfamily *Orthocoronavirinae*, family *Coronaviridae*, order *Nidovirales* and kingdom *Riboviria*. It is characterised by single-stranded RNA genome, positive direction and a helically symmetric nucleocapsid^(12)^. According to their genetic properties, coronaviruses are grouped into four genera: *α*-CoV, *β*-CoV, *γ*-CoV and *δ*-CoV, SARS-CoV-2 belonging to *β*-CoV (7). Another important aspect concerning the characteristics of coronaviruses is their structure, comprising at least four proteins: *Spike* (S), Envelope (E), Membrane (M) and nucleocapsid (N)^(13)^.

It is worth mentioning that protein S mediates SARS-CoV-2 binding to receptors on the surface of the host cell, resulting in fusion and subsequent viral entry^(13,14)^. Moreover, the protein S binding domain binds to the peptidase domain of the human angiotensin-converting enzyme 2, which acts as a virus receptor for SARS-CoV-2, enabling it to enter the host cell. This enzyme is a membrane protein expressed in several cells, like cells in the alveolar epithelium, trachea and bronchi, serous bronchial glands, as well as alveolar monocytes, macrophages, and pneumocytes^(15)^.

The SARS-CoV-2 binding to angiotensin-converting enzyme 2 receptors triggers conformational changes in protein S, inducing its cleavage by transmembrane serine protease 2. After that, the virus is transported to the cytoplasm through endocytosis. The low pH inside the endosomes induces the activity of the host protease cathepsin-L, which cleaves protein S. The cleavage of protein S induces fusion of viral envelope and endosomal phospholipid membrane to release the viral genomic RNA from the positive strand in cell cytoplasm^(7)^.

In addition, the RNA-dependent viral RNA polymerase (RdRp), enzyme encoded in the SARS-CoV-2 genome, is essential for virus replicative cycle. Initially, the polyprotein precursor is formed from which the RdRp-containing subunit is proteolytically cleaved. Subsequently, RdRp is integrated into a membrane-associated viral enzyme complex that drives the synthesis of negative chain RNA. The negative RNA strand is used as a template for viral mRNA synthesis. It is noteworthy that the RdRp enzyme has a deep groove as an active site for RNA polymerisation^(7)^.

Possible antiviral therapies could be classified into two categories, depending on the target, either acting against the coronavirus itself or protecting the immune system^(14)^. The therapies against the coronavirus involve the inhibition of viral RNA synthesis by affecting the viral genetic material, inhibition of viral replication and blocking viral enzymatic activity. In addition, certain therapies are based on blocking of virus interaction with human cell receptors or of the viral self-assembly process, through affecting structural proteins^(14)^.

Several *in vitro* studies have evaluated the effectiveness of Zn as an antiviral agent. Although the involved mechanisms are not understood well enough in detail, the mechanism of antiviral activity of Zn are reportedly specific to each viral type, and Zn ion availability appears to play a significant role in its antiviral efficacy^(16)^. In addition, Zn deficiency has been associated with increased sensitivity to infectious diseases, including viral infections^(17,18)^.

Antiviral functions of Zn are based on inhibition of physical processes, as virus fixation, infection and coating, as well as the inhibition of viral protease and polymerase enzymatic function. The increase in the intracellular Zn concentrations could interfere with the proteolytic processing of viral polyprotein, influencing its enveloping. Furthermore, high intracellular Zn concentrations may affect directly the viral protease (picornavirus, encephalomyocarditis and polioviruses), and to alter the tertiary structure of the protein, as in the case of the encephalomyocarditis virus. In addition, Zn inhibits viral and host cellular membrane fusion, preventing viral infection^(12,19–21)^.

Another mechanism underlying antiviral activity of Zn refers to its ability to dose-dependently inhibit angiotensin-converting enzyme 2 enzymatic activity, that is, the higher the Zn concentration, the greater the enzymatic inhibition efficiency. Thus, the researchers suggest that Zn might inhibit the interaction between SARS-COV-2 protein S and angiotensin-converting enzyme 2, a recipient of the enveloped virus^(15)^.

Ionophores, such as hydroxychloroquine, hinokitiol, pyrrolidine dithiocarbamate and viral pyrithione, play an important role in the antiviral activity of Zn, stimulating Zn influx into cellular cytoplasm^(12,16,19)^. Cell culture studies revealed that high concentrations of Zn and the supplementation of compounds that stimulate the cellular Zn influx inhibited the replication of several RNA viruses, including the influenza virus, respiratory syncytial virus and various picornaviruses^(12,16)^.

Zn cations, especially in combination with the ionophore pyrithione, reportedly inhibit SARS-COV-2 RNA polymerase activity, reducing viral replication. In addition, Zn inhibits the activity of the RdRp enzyme of SARS-CoV-2 also during the elongation phase of RNA synthesis. Thus, Zn ions appear to inhibit adequate proteolytic processing of replicase polyproteins and RdRp activity^(9,16)^.

Concerning the efficacy of chloroquine ionophore againt SARS-CoV-2, the results show that chloroquine increases the flow of Zn into cells. Derwand R and Scholz^(22)^ supplemented Zn associated with chloroquine and hydroxychloroquine during the treatment of COVID-19, and they found an increase in intracellular Zn concentrations, mainly in lysosomes. In addition, it was also observed that the higher the intracellular concentration of Zn, the greater its ability to inhibit the RdRp enzyme and, consequently, the intracellular replication of SARS-CoV-2. This potentially improved clinical outcomes of patients with COVID-19 treated with these drugs^(22)^.

**Fig. 2.**
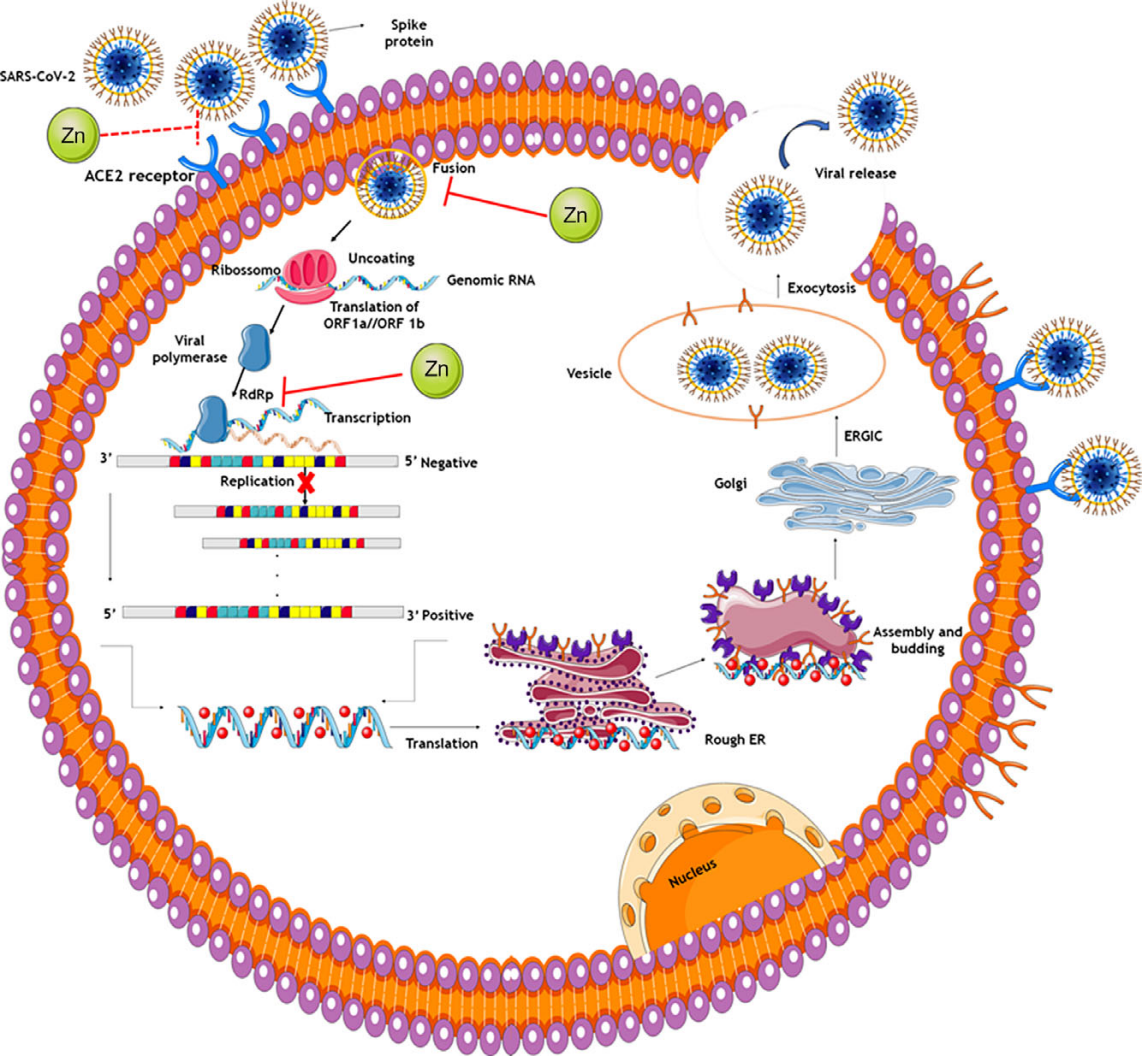
Potential mechanisms of zinc in COVID-19 therapy. The spike proteins of SARS-CoV-2 bind to ACE2 receptors. The virion then releases the RNA genome into the cell and translation of structural and non-structural proteins follows. ORF1a and ORF1ab are translated to produce pp1a and pp1ab polyproteins, which are cleaved by the proteases that are encoded by ORF1a to yield non-structural proteins. This is followed by assembly and budding into the lumen of the ERGIC. Virions are then released from the infected cell through exocytosis. Zinc might also possess antiviral activity through inhibition of RdRp and reduction in template binding. Indirect evidence also indicates that zinc might decrease ACE2 activity, known to be the receptor for SARS-CoV-2, contributing to inhibit the fusion of SARS-CoV-2 in the cell membrane. ACE2: angiotensin-converting enzyme 2; Rough ER: rough endoplasmic reticulum; ERGIC: endoplasmic reticulum–Golgi intermediate compartment.

The antiviral activity of Zn could also be exerted through metallothioneins, low-molecular-weight enzymes that bind and transport Zn. Read *et al.* ^(23)^ demonstrated that the induction of these enzymes, particularly of subfamily 1 and 2 members, could inhibit the replication of the hepatitis C virus, and that the Zn antiviral activity could be mediated by mechanisms that involve metallothioneins. Figure 2 shows potential mechanisms underlying activity of Zn in therapy for COVID-19.

### Immunomodulatory zinc activity

COVID-19 predominantly affects the respiratory system, resulting in pneumonia and acute respiratory distress syndrome, leading the need for mechanical ventilation. Advanced age, acute respiratory distress syndrome, need for mechanical ventilation and impaired immune system are related to higher COVID-19 mortality^(9)^. Zn acts as an important immunomodulator, as it regulates the proliferation, differentiation, maturation, and function of leucocytes and lymphocytes, and it also modulates the inflammatory response. In addition, Zn supplementation in adults and children has a beneficial effect to reducing virus-induced symptoms and illness time, such as colds and flu^(24,25)^.

In acute phase response to infection, a systemic response coordinated by cytokines decreases concentrations of trace elements in plasma, including Zn. This is due to the fact that some pathogens need minerals for their growth, which contributes to reducing the levels of these minerals in plasma^(26)^. During infection, polymorphonuclear leucocytes migrate by adhesion and chemotaxis to the infected tissue in response to inflammatory signals. As a defence mechanism, the body uses alternatives to maintain homoeostasis, inducing phagocytosis, and subsequently, increased production of reactive oxygen species, mediated mainly by the high activity of NADPH oxidase^(26,27)^.

One of the hallmarks of COVID-19 is an imbalanced immune response due to hyperinflammation, including a very rapid and enhanced production of proinflammatory cytokines. During inflammatory response, Zn is redistributed to the tissues, resulting in serum hypozincemia. Thus, subjects with COVID-19 are at risk of Zn deficiency. Furthermore, in combination with the pre-existing suboptimal Zn supply, this will decrease serum Zn levels to critically low values and thereby significantly increase the susceptibility for co-infections with detrimental progression^(28,29)^.

Zn deficiency reduces the antibody production and impairs the innate immune system because of reduced natural killer cell activity, impaired monocyte cytokines production, impaired chemotaxis and oxidative explosion of neutrophilic granulocytes^(12,30)^. Zn deficiency could also induce thymus atrophy, inducing altered production of thymic hormones, lymphopenia, cellular defects and antibody-mediated responses that trigger increased infection rates and duration. This is because Zn deficiency reduces the number of peripheral and thymic T cells, their proliferation in response to phytohemagglutinin, and the functions of T cell cytotoxic auxiliaries^(12,30)^.

Associated with this, Zn deficiency acts indirectly, reducing the levels of active serum thymulin, a Zn-dependent hormone that regulates the differentiation of immature T cells in the thymus and the function of mature peripheral T cells^(12,30)^. Most antigens are dependent on T cells and, therefore, during Zn deficiency, the production of antigens is compromised, and the body becomes unable to respond with the synthesis of antibodies in response to neoantigens^(31)^.

Zn deficiency also could impair the immune system because Zn induces the production of interferon-*α* and interferon-*γ* by leucocytes, enhancing its antiviral activity^(31)^. It induces cellular resistance to apoptosis by inhibiting caspase 3, 6, and 9 and increases the Bcl-2/Bax ratio, which could contribute to increasing T cell amounts^(32)^. Thus, Zn contributes to enhance immune response to viral infection^(12,30)^.

Moreover, Zn is essential for the barrier function of mucosal epithelium due to its antioxidant and anti-inflammatory activity. Zn also regulates tight junction proteins that are important for the maintenance of mucosal membrane integrity. However, reduction of mucosal integrity and loss of tight junction cohesion aggravate viral inflammation^(33,34)^.

**Fig. 3.**
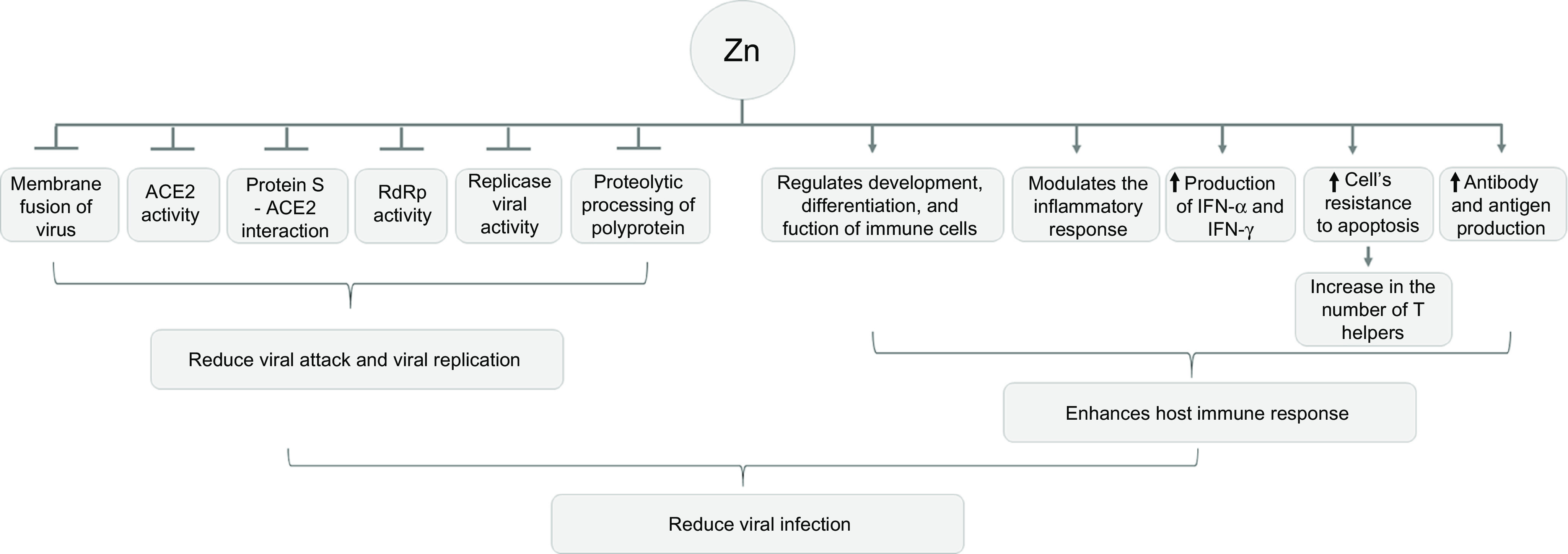
Antiviral and immunomodulator effects of zinc. ACE: angiotensin-converting enzyme; IFN: interferon.

Figure 3 shows the possible antiviral and immunomodulatory effects of Zn.

### Zinc supplementation studies

Studies of Zn supplementation have been done to evaluate immunomodulatory Zn activity and antiviral Zn action. Hasegawa *et al.* ^(35)^ examined the ability of human neutrophils to produce reactive oxygen species to investigate the effects of Zn on non-specific immune functions. They stimulated neutrophils with opsonised zymosan and phorbol myristate acetate in presence of 1–10^–3^ mmol/l of Zn chloride *in vitro*. Their results suggested that Zn activates protein kinase C and promotes myeloperoxidase degranulation, suggesting that Zn supplementation beyond physiological doses improves neutrophil functional activity. Thus, the authors suggested that Zn is essential for optimal functioning of non-specific immunity.

In order to better understand the Zn activity on thymic function and immune homoeostasis, Lovino, Mazziotta and Carulli^(36)^ conducted a prospective clinical study using high doses of oral Zn (600 mg/d zinc sulphate heptahydrate) to improve immune reconstitution after haematopoietic stem cell transplantation. Patients received Zn supplementation by postoperative day 5 to day 100 after the transplantation. The authors found an increase in CD4+ lymphocytes and T-lymphocyte receptor only in the group treated with Zn, and they concluded that high-dose Zn supplementation is safe and could improve thymic reconstitution after haematopoietic stem cell transplantation.

Mariani *et al.* ^(37)^ evaluated the Zn activity in modulating the immune response, in balance of T helper 1 and 2 cells and in low-grade systemic inflammation during ageing. Researchers found that the supplementation with 10 mg/d of zinc aspartate for 48 d increased the Zn concentration in blood, and it increased plasma IL-6, monocyte chemotactic protein 1 and lytic activity of natural killer cells in elderly individuals with Zn plasma concentrations below 11 μmol/l.

In a study by Ganatra *et al.* ^(38)^, supplementation with 10 mg/kg of zinc gluconate solution was able to suppress neutrophil recruitment, inflammatory response and subsequent lung injury after polymicrobial sepsis in rats. In addition, this study mentioned that Zn had modulating effects of the inflammatory cascade, proved by low serum concentrations of IL-2, IL-6 and IL-1*β*, reducing hyperinflammatory response to infectious agents.

Zn deficiency is also associated with increased susceptibility to infectious diseases due to activity of several pathogens, including viruses^(39,40)^. In such cases, Zn administration at sufficient therapeutic doses has the potential to improve or restore immune cell function^(12)^. Mossad *et al.* ^(41)^ tested the effectiveness of zinc gluconate in reducing the duration of symptoms caused by common cold in 100 individuals. Patients received one lozenge every 2 h while awake, containing 13·3 mg of Zn in the form of zinc gluconate, as long as they had cold symptoms. The main results found were less time to complete the resolution of symptoms, fewer days with cough, headache and hoarseness in the group supplemented with Zn.

A study conducted by Sempértegui *et al.* ^(42)^ aimed to evaluate how Zn supplementation affect the respiratory tract disease, immunity and growth in malnourished children. It demonstrated that zinc sulphate supplementation of 10 mg/d for 8 weeks reduced the incidence of fever, cough and respiratory tract secretions, reinforcing the importance of Zn in immunity against respiratory infections.

Results by Suara *et al.* ^(43)^ research suggest that antiviral activity of Zn against the respiratory syncytial virus, the main cause of paediatric lower respiratory tract disease. Authors verified *in vitro* inhibitory effect of three Zn salts (acetate, lactate and zinc sulphate) on respiratory syncytial virus replication at concentrations ranging from 1 to 10 mM or 10 to 100 μM, and they found that the inhibitory effect of Zn salts was dependent on its concentration, especially Zn in the form of sulphate.

In the context of morbidity and mortality due to COVID-19, Zn deficiency might be relevant to the negative outcome in patients with severe disease, including elderly patients with hypertension, diabetes mellitus, CHD or chronic obstructive pulmonary disease^(25)^. It has also been demonstrated that Zn could act synergistically when co-administered with standard antiviral therapy^(39)^.

Velthuis *et al.* ^(16)^ demonstrated that the combination of Zn with its ionophore pyrithione, at low concentrations (2 µM Zn2 + and 2 µM pyrithione), inhibited the replication of SARS-coronavirus (SARS-CoV) and equine arteritis virus in cell culture. This study also showed that an activity assay for the multiprotein replication and transcription complex, isolated from the cells infected with SARS-CoV or equine arteritis virus, allowed to eliminate the need to use pyrithione to transport Zn across the plasma membrane, and that Zn efficiently inhibited the RNA synthesis activity of the replication and transcription complex of both viruses.

In the case report by Finzi^(6)^, including four patients diagnosed with SARS-CoV-2, one male and three females, aged between 26 and 63 years, patients were supplemented with Zn in combination with hydroxychloroquine. In that study, two participants were supplemented with zinc citrate tablets (23 mg of elemental Zn), a patient with zinc citrate/zinc gluconate (23 mg) and a patient with zinc acetate (15 mg), and individuals started Zn therapy at different times during the course of the disease. All participants exhibited significant improvement in the symptoms of the disease after a day of therapy using high doses of Zn associated with the drug, suggesting that Zn therapy has a beneficial effect on clinical recovery.

In order to investigate the role of Zn in elderly patients hospitalised with COVID-19, Yao *et al.* ^(18)^ carried out a retrospective study to evaluate the survival of patients treated with 440 mg of zinc sulphate (100 mg of elemental Zn). Results obtained from the study did not reveal an association between Zn supplementation and the survival rate in the group of elderly people evaluated.

In this perspective, Derwad *et al.* ^(44)^ evaluated the effect of early supplementation with 220 mg of zinc sulphate (50 mg of elemental Zn) and other associated drugs. There was a significant reduction in hospitalisation and mortality in patients undergoing triple intervention (Zn plus low-dose hydroxychloroquine and azithromycin). However, more research is necessary to demonstrate the efficacy of Zn supplementation to increase survival in patients with COVID-19.

Table 2 shows studies that evaluated the effect of Zn supplementation associated with therapeutic drugs in patients with confirmed COVID-19.

**Table 2.** Studies that evaluated the effect of zinc supplementation associated with therapeutic drugs in patients with confirmed COVID-19

Article	Methodology	Results
Velthuis *et al.* ^(16)^	2 µM Zn2 + and 2 µM pyrithione. Cell culture	Inhibited SARS-CoV replication.
Finzi^(6)^	*n* 4. Group A (*n* 2): 23 mg elemental Zn. Group B (*n* 1): 23 mg zinc citrate/zinc gluconate. Group C (*n* 1): 15 mg zinc acetate	Significant improvement in symptoms of COVID-19 after one day of therapy using high doses of Zn associated with hydroxychloroquine.
Yao *et al.* ^(18)^	*n* 196 elderly patients. Dose: 440 mg of zinc sulphate (100 mg of elemental Zn)	No association between Zn supplementation and survival rate in elderly people.
Carlucci *et al.* ^(45)^	*n* 411. Drugs + 220 mg of zinc sulphate (50 mg of elemental zinc). Treatment: 5 d	Decreased: mortality, need for ICU care, need for ventilation. High probability of returning home.
Derwand; Scholz; Zelenko^(44)^	*n* 141. Drugs + 220 mg of zinc sulphate (50 mg of elemental zinc). Treatment: 5 d	Significantly fewer hospitalisations.
Sattar *et al.* ^(46)^	*n* 3. Drugs + 220 mg of zinc sulphate (50 mg of elemental zinc). Treatment: 5 d	All patients were recovered.

Although studies have already shown the benefits of Zn in infections and respiratory diseases, beneficial effects of Zn supplementation in patients with COVID-19 and the definition of dosage and use duration are still being consolidated. Thus, considering the role in immune function and the potential to decrease coronavirus replication, Zn has been extensively investigated as a treatment strategy for patients with COVID-19^(45–47)^.

On the other hand, Zn supplementation may not be useful in the conditions of Zn sufficiency^(48,49)^. Results of studies that investigated the effects of Zn supplementation on patients with infection with the HIV, for example, are contradictory because the different Zn status of the patients. They suggest that while moderate Zn supplementation to Zn-deficient subjects can advance their immune responses, it may have harmful effects when given to Zn-sufficient ones^(39)^. This fact underlines the potential benefits of monitoring the Zn status of the patients with viral infections like COVID-19.

Excess dietary Zn could also impair immune response by inhibiting T-lymphocyte and B-lymphocyte function, reducing intracellular pathogen destruction in macrophages or inducing an overload of regulatory T cells. Therefore, a balanced Zn homoeostasis is critical for adequate immune functions^(34)^. Similarly, Matwald, Rink^(50)^ reported that Zn supplementation excess was able to reduce the expression of interferon-*γ*, reducing the expression of the interferon 1 regulatory factor in regulatory T cells. Thus, the reduction of the expression of this cytokine results in immunological impairment, since it reduces the defence capacity against pathogens with high viral activity.

Studies that evaluate the effect of supplementation dietary Zn on non-specific immunity are controversial. Morgan *et al.* ^(51)^ reported that supplementation of zinc gluconate is able to reduce the infiltration of neutrophils into the airways and the release of TNF-*α* by inhibiting NF-kB-dependent transcription of inflammatory genes, enhancing its antiviral activity and inflammation. In contrast, Wessels, Maywald and Rink^(28)^ explain that excessive doses of Zn could affect the immune response, because brings about overload of regulatory T cells, direct activations of macrophages, and suppression of T and B cell function, reducing the immune response to viral infection.

Furthermore, acute exposure to high doses of Zn could induce disorders of the gastrointestinal tract, including nausea, vomiting, loss of appetite, epigastric pain, diarrhoea, in addition to headache and fatigue. Chronic Zn toxicity could include lethargy, Cu deficiency and severe Fe deficiency anaemia. Excessive Zn levels are cytotoxic and have been shown to induce higher mortality in experimental studies. The risk of developing adverse effects can limit tolerance and long-term use of Zn^(52)^.

Researches that evaluate Zn supplementation as a therapeutic strategy and prophylaxis in groups at risk for COVID-19 are necessary because Zn is an economical option and simple to use. Some clinical trials on Zn supplementation alone and in combination with other drugs such as chloroquine have already been registered. It is important to highlight that the COVID-19 Treatment Guidelines^(53)^ recommend against using Zn supplementation above the RDA for the prevention of COVID-19, except in a clinical trial.

## Conclusion

Literature shows the role of Zn as an essential mineral to several biological functions in the body, its action as an immunomodulator nutrient and its antiviral activity. Thus, although there is still a lack of studies evaluating Zn supplementation in patients with COVID-19, the results on the topic show the need to control Zn deficiency, as well as maintaining its homoeostasis in the body in order to strengthen the immune system and improve the prevention of highly complex viral infections, such as that of the COVID-19.
